# Experimental Study on Bioluminescence Tomography with Multimodality Fusion

**DOI:** 10.1155/2007/86741

**Published:** 2007-09-13

**Authors:** Yujie Lv, Jie Tian, Wenxiang Cong, Ge Wang

**Affiliations:** ^1^ Medical Image Processing Group, Institute of Automation, Chinese Academy of Sciences, P.O. Box 2728, Beijing 100080, China; ^2^ Life Science Center, Xidian University, Xian, Shaanxi 710071, China; ^3^ Division of Biomedical Imaging, VT-WFU School of Biomedical Engineering and Sciences, Virginia Polytechnic Institute and State University, Blacksburg, VA 24061, USA

## Abstract

To verify the influence of a priori information on the nonuniqueness problem of bioluminescence tomography (BLT), the multimodality imaging fusion based BLT experiment is performed by multiview noncontact detection mode, which incorporates the anatomical information obtained by the microCT scanner and the background optical properties based on diffuse reflectance measurements. In the reconstruction procedure, the utilization of adaptive finite element methods (FEMs) and a priori permissible source region refines the reconstructed results and improves numerical robustness and efficiency. The comparison between the absence and employment of a priori information shows that multimodality imaging fusion is essential to quantitative BLT reconstruction.

Bioluminescence tomography (BLT) has an increasingly significant effect on revealing the molecular and cellular information in vivo [1]. When the bioluminescence imaging experiment is performed, luciferase can be introduced into various types of cells, organisms, and genes in a living mouse. Then, luciferin is combined with luciferase in the presence of oxygen and ATP to generate bioluminescent signals of about 600 nm in wavelength. The mechanism of BLT is to identify bioluminescent source from the light flux detected on the surface of small animal. However, three-dimensional bioluminescent source reconstruction is an inverse source problem in theory, which has been less researched and is different from the inverse scattering imaging, such as diffusion optical tomography (DOT). Then, in the highly heterogeneous biological tissues, the scattering and absorption of the photons emitted by bioluminescent source further increase the difficulty of source localization. In addition, although the absence of external illumination sources acquires high-sensitive signal and yields high-contrast image in bioluminescence imaging, it complicates the tomographic problem. Therefore, the unique and quantitative reconstruction of bioluminescent source is a topic of further investigation [2].

Based on diffusion approximation theory, the uniqueness theorem indicates that it is necessary to utilize a priori information to solve the nonunique problem of BLT [3]. In view of the spectral characteristics of the underlying bioluminescent source, hyper- and multispectral BLT methods are proposed [4, 5, 6, 7]. Taking into account the surface light power distribution and the heterogeneous structure of the phantom, a priori permissible source region based BLT reconstruction method is developed on the fixed discretized mesh [8]. Then, the multilevel adaptive finite element based tomographic algorithm is also developed, which further reduces the ill-posedness of BLT and improves the reconstruction quality [9]. In this research, a BLT experiment is performed, which incorporates the anatomical and background optical information. Using our proposed tomographic algorithm [9], the reconstructed results show that multimodality imaging fusion is indispensable to quantitative BLT reconstruction.

Figure 1(a) shows the BLT prototype for bioluminescence imaging. The main component of the equipment is a cooled CCD camera (Princeton Instruments, USA), which collects optical signals emitted from bioluminescent source in the phantom. The combination of the vertically rotated stage under computer control and the camera realizes the multiview noncontact detection. When the phantom is placed on the stage, we may manually adjust the distance between the lens and the phantom surface through the controlled transport for the best signal acquisition. In addition, the utilization of light-tight enclosure guarantees that the bioluminescence imaging is performed in a totally dark environment.

When bioluminescent photons propagate in biological tissue, the radiative transfer equation (RTE) may precisely describe photon transportation. Diffusion equation as an approximation has been extensively applied in terms of high scattering characteristic of tissues [8]. In addition, Robin boundary condition is used to deal with the refractive indices mismatch between the small animal and the external medium [8]. In the framework of adaptive finite element analysis, a linear relationship between the measurable boundary flux $\Phi_{k}^{m}$ and the unknown source density $S_{k}^{p}$ can be established on the kth discretized level in terms of a priori permissible source region [9]:

(1) $$A_{k}S_{k}^{p}=\Phi_{k}^{m}.$$

Then, we define the following kth level minimization problem to reconstruct source distribution based on Tikhonov regularization methods:

(2) $$\underset{S_{\text{inf}}^{k}\leq S_{k}^{p}\leq S_{\text{sup}}^{k}}{\text{min}}\Theta_{k}\left(S_{k}^{p}\right)=\{\|A_{k}S_{k}^{p}-\Phi_{k}^{m}{\|}_{\Lambda }+\lambda_{k}\eta_{k}(S_{k}^{p})\},$$

where $S_{\text{inf}}^{k}$ and $S_{\text{sup}}^{k}$ are the kth level lower and upper bounds of source density; Λ is the weight matrix, $\|V{\|}_{\Lambda }=V^{T}\Lambda V$; $\lambda_{k}$ the regularization parameter; and $\eta_{k}\left(\cdot \right)$ the penalty function. Through selecting the effective optimization method, we can obtain the preferable BLT reconstruction.

In this bioluminescence imaging experiment, a heterogeneous physical phantom of 30 mm height and 15 mm radius is designed and fabricated. The phantom, shown in Figure 1(b), is made up of four different materials, that is, high-density polyethylene (8624K16), nylon 6/6 (8538K23), delrin (8579K21), and polypropylene (8658K11) to represent muscle, lungs, heart, and bone, respectively. Two luminescent sources of about 1.9 mm height and 0.56 mm diameter are embedded in the left-lung region of the phantom with the centers at (−9.0, 1.5, 0.0) and (−9.0, −1.5, 0.0). Their source densities are 155.53 nW/mm^3^ and 178.49 nW/mm^3^, respectively. The slice of the phantom representing anatomical information is obtained by microCT scanner for generating the volumetric finite element mesh, as shown in Figure 1(c). In addition, the optical properties of four materials as a priori information need to be acquired. To each material, a cylindrical phantom with 10 mm radius and 20 mm height was made. The side surface of the phantom was blackened. After the stable light was obtained by an integrating sphere, it was guided for illumination through the optic fiber. The optic fiber was inserted into a small hole of 10 mm depth at the center of the phantom bottom surface. The CCD camera was used to detect the output photon density on the other bottom surface of the phantom. After the data acquisition, an optical tomography procedure was used to decide the optical parameters of each material. Specifically, the specimen was considered as a semi-infinite homogeneous medium, and diffusion theory was applied with the extrapolated boundary condition. The photon density on the bottom surface was predicted by an analytic formula; and then, the absorption and reduced scattering coefficients were calculated by a nonlinear least-square fitting, as shown in Table 1. The detailed information can be found in [8]. In the noncontact detection mode, multiview detection is essential to reduce the influence of the curved surface of the phantom on the measured value. In this experiment, four views are acquired, which are separated by 90 degrees along radial directions. Its schematic diagram is displayed in Figure 2. Measured data on the CCD camera is transformed from the recorded pixel gray levels by $\varphi =\text{pix}\times 0.377\;\text{pW}/{\text{mm}}^{2}$ [8], where φ is the photon density and pix denotes the pixel value.

When the BLT reconstruction is performed, the physical phantom is an anatomical and optical homogeneous object if two types of a priori information are not considered. Area-weighted method is employed to approximate the homogeneous optical property. Through the difference of detected surface light power distribution in four views, as demonstrated in Figure 3 [8], we may infer the permissible source region as $P_{s}=\left\{\left(x,y,z\right)|x<0,\;-1.5<z<1.5,\;\left(x,y,z\right)\in \text{the phantom}\right\}$, as shown in Figure 4(a). During the reconstruction procedure, a modified Newton method with active-set strategy is employed for the minimization problem $\Theta_{k}\left(S_{k}^{p}\right)$ at each level. Using a posteriori error estimation techniques, the elements with higher errors and reconstructed values in the forbidden and permissible source regions, respectively, are selected for adaptive mesh refinement after the reconstruction is accomplished on the coarse mesh. *Red-green* refinement strategy reasonably implements the local mesh refinement. Note that BLT with a coarsely discretized mesh means less unknown variables, higher computational efficiency, and better numerical stability than that with a finely discretized counterpart. Hence, the optimization of the objective function $\Theta_{k}\left(S_{k}^{p}\right)$ is indispensable on the coarse mesh. The detailed explanation and discussion can be found elsewhere [9]. After four mesh refinements, Figure 5(a) shows the final reconstructed results. Due to the absence of anatomical and optical information, the BLT reconstruction cannot distinguish two light sources, and the reconstructed position is also far from the actual one despite that the roughly inferred permissible source region is utilized. When the anatomical information is considered, the selection of permissible source region may be restricted in the left lung, as illustrated in Figure 4(b). Two light sources can be distinguished from the reconstructed results, as shown in Figure 5(b). Although there are small relative errors in source density between the reconstructed and actual sources, the preferable source localization cannot be obtained. Finally, Figure 5(c) displays the reconstructed results in terms of the utilization of anatomical and optical information. The position and density of light sources are better reconstructed. The quantitative comparison above is summarized in Table 2, which further demonstrates the importance of anatomical and optical information for BLT reconstruction.

In this research, to our knowledge, we have first presented that multimodality imaging fusion is essential for quantitative BLT reconstruction through the experimental comparison based on the multilevel adaptive finite element algorithm. Despite that there is a linear relationship between the boundary measured data and the unknown source variables, inherent characteristics make BLT reconstruction more ill-posed compared with fluorescence imaging. The more a priori knowledge we have, the better the light source is reconstructed [3]. The utilization of anatomical and optical information not only approaches the basal optical transportation model better, but also helps to infer the permissible source region. When the small animal-based BLT equipment and algorithm researches are ongoing for the practical application to biology, this research provides the basically experimental verification for BLT reconstruction. 

## Figures and Tables

**Figure 1:**
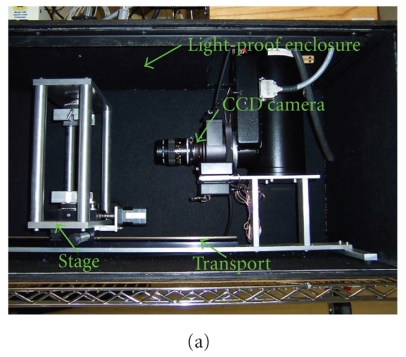

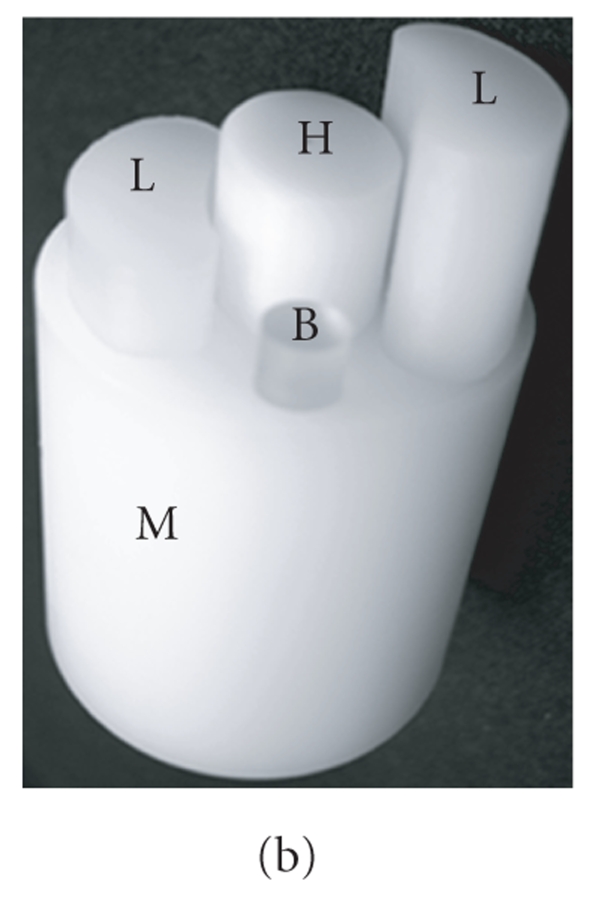

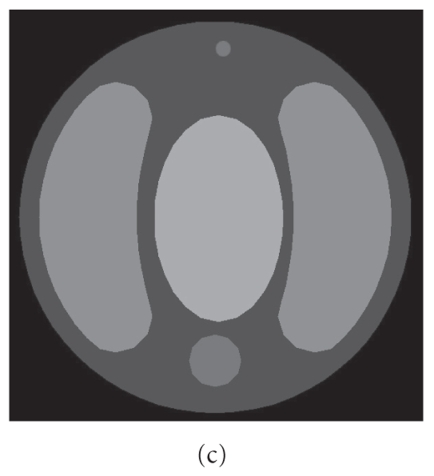
BLT system and physical phantom. (a) Multiview noncontact BLT prototype; (b) the physical heterogeneous phantom consisting of bone (B), heart (H), lungs (L), and muscle (M); and (c) a slice scanned by microCT scanner.

**Figure 2:**
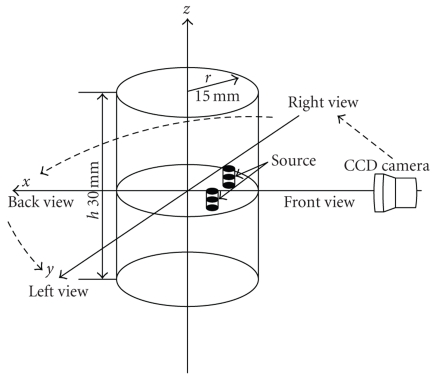
Schematic diagram of the multipleview bioluminescence imaging experiment.

**Figure 3:**
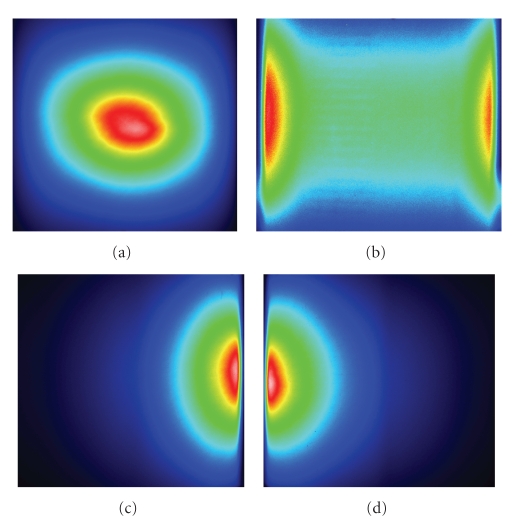
The detected photon energy distribution on the phantom surface by CCD camera.

**Figure 4:**
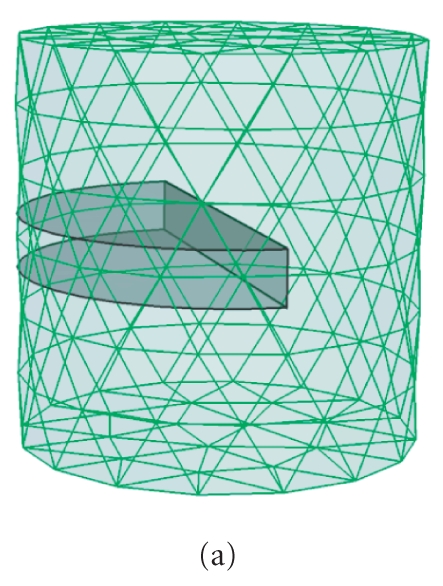

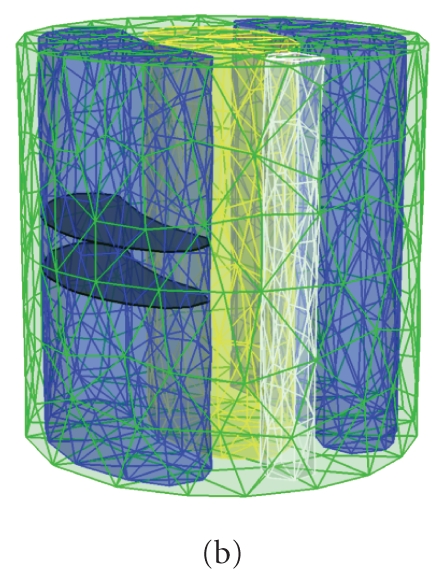
The initial homogeneous (a) and heterogeneous (b) finite element meshes used in the BLT reconstruction. The black areas represent a priori permissible source regions.

**Figure 5:**
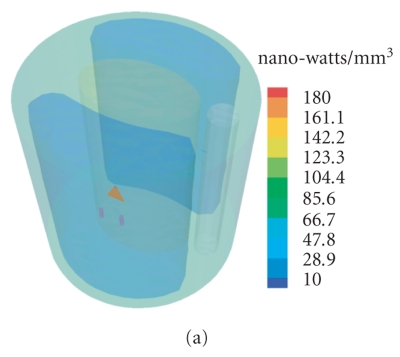

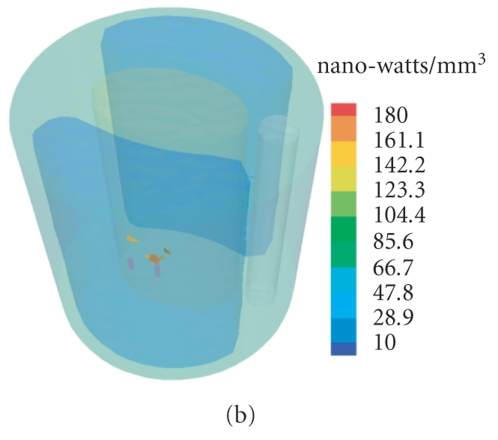

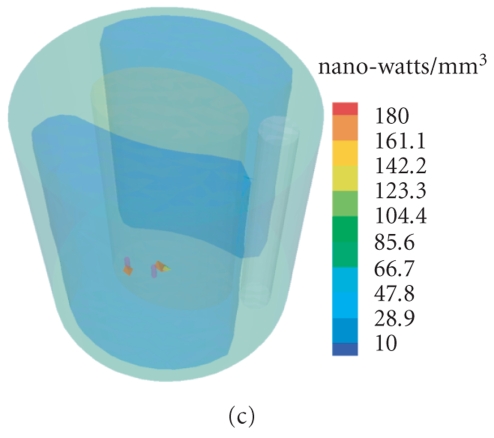
Comparison between the actual and reconstructed sources. (a) The BLT reconstruction without anatomical and optical information; (b) the counterpart only with anatomical information; and (c) that with anatomical and optical information.

**Table 1:** Optical properties of the physical heterogeneous phantom.

Material	Muscle	Lung	Heart	Bone
$\mu_{a}\left[{\text{mm}}^{-1}\right]$	0.007	0.023	0.011	0.001
$\mu_{s}^{\prime }\left[{\text{mm}}^{-1}\right]$	1.031	2.000	1.096	0.060

**Table 2:** Quantitative comparison between the reconstructed and actual sources with and without a priori anatomical and optical information. Density errors are calculated by $|S_{\text{recons}}-S_{\text{real}}|/S_{\text{real}}$.

No.	Recons. pos.	Recons. dens.	Pos./dens.
	(mm)	(nW/mm^3^)	errors
1	(−6.39,0.39,1.31)	168.80	N.A.
2	(−5.83,2.85,−0.09)	143.69	3.45/7.61
	(−6.05,-1.31,0.10)	177.26	2.96/0.69
3	(−9.40,1.31,−0.26)	167.49	0.51/7.69
	(−8.11,−1.76,0.24)	175.74	0.96/1.54
